# New diagnostic SNP molecular markers for the *Mytilus* species complex

**DOI:** 10.1371/journal.pone.0200654

**Published:** 2018-07-12

**Authors:** Joanna Wilson, Iveta Matejusova, Rebecca E. McIntosh, Stefano Carboni, Michaël Bekaert

**Affiliations:** 1 Institute of Aquaculture, Faculty of Natural Sciences, University of Stirling, Stirling, Scotland, United Kingdom; 2 Marine Scotland Science, Aberdeen, Scotland, United Kingdom; National Cheng Kung University, TAIWAN

## Abstract

The development of diagnostic markers has been a long-standing interest of population geneticists as it allows clarification of taxonomic uncertainties. Historically, there has been much debate on the taxonomic status of species belonging to the *Mytilus* species complex (*M*. *edulis*, *M*. *galloprovincialis* and *M*. *trossulus*), and whether they are discrete species. We analysed reference pure specimens of *M*. *edulis*, *M*. *galloprovincialis* and *M*. *trossulus*, using Restriction site associated DNA (RAD) sequencing and identified over 6,000 SNP markers separating the three species unambiguously. We developed a panel of diagnostic SNP markers for the genotyping of *Mytilus* species complex as well as the identification of hybrids and interspecies introgression events in *Mytilus* species. We validated a panel of twelve diagnostic SNP markers which can be used for species genotyping. Being able to accurately identify species and hybrids within the *Mytilus* species complex is important for the selective mussel stock management, the exclusion of invasive species, basic physiology and bio-diversity studies.

## Introduction

Blue mussel (*Mytilus edulis*, Linneaus, 1758) has been integral part of humans’ diet for millennia, their shells have been found in middens dated back to the late Mesolithic periods, 5,000 B.C. [1]. Today, Europe is a major contributor to mussel’s production, supplying over a third of the total commercial outputs. Aquaculture is by far the main source of this commodity and it is responsible for over 90 percent of total landings. *M*. *edulis* and *Mytilus galloprovincialis* (Lamarck, 1819) are the two main species cultivated in Europe with an output of 550,000 tonnes and € 900 million per year [2]. These two species, together with the Baltic mussel (*Mytilus trossulus*, Gould, 1850), belong to the “*Mytilus* species complex”. Hybridisation between these species has been observed across the world; *e*.*g*., in the Pacific Ocean [3–6], in the Irish Sea [7–9] and Scotland where all combinations of species hybrids have been identified [10–13]. *M*. *trossulus* has been often associated with lower meat yield, thinner shell and reduced shelf life compared with *M*. *edulis* [10,14] and it is therefore considered undesirable within the European aquaculture context. Being able to accurately identify species and hybrids within the *Mytilus* species complex is therefore important for the management of a potentially economically damaging species.

Hybridisation and introgression between species are common evolutionary phenomena [15–17]. Introgression arises from repeated backcrossing with fertile hybrids, allowing stable integration of genomic material of one species into the genome of another species without a significant deleterious effect on fitness [18]. Accurate identification of species (including cryptic or complex species) is important from commercial, conservation and research viewpoints and can be significantly impaired when hybridisation or introgression occur.

There are some distinguishing morphological features that could be employed for marine mussel species (*Mytilus* spp.) identification, such as shell colour, shape, texture and size [19–21]. However, a range of biotic and abiotic factors, including hydrodynamic conditions [22], water temperature and salinity [23], can affect these features making individuals often morphologically similar, especially in sympatric populations [24–26].

The development of diagnostic markers has been a long-standing interest of population geneticists as it allows clarification of taxonomic uncertainties. The first tools used to distinguish between mussel species were allozymes. Numerous allozyme markers have been developed and used for *Mytilus* species identification [10,14,23,27]. Nonetheless, low as well as high variation between individual of the *Mytilus* complex render the technique only marginally more useful than the identification by morphological features [28]. Over the past three decades, a range of species diagnostic markers have been developed for single locus genotyping of *Mytilus* species of which the most routinely used is the nuclear DNA marker Me15/16 [29]. Genotyping with the Me15/16 is favoured due to its simple methodology of PCR amplification and identification of a size-specific gene fragments unique for each of the *Mytilus* species [30]. Single locus genotyping delivers more accurate identification of *Mytilus* species. than studying morphology or allozymes, but has limited potential for analysing patterns of hybridisation or genome introgression [17]. Multilocus genotyping, using genome wide panels of single nucleotide polymorphism (SNP) markers by comparison, allows for a far better understanding of introgression [31–35]. There are only few studies on multilocus genotyping in Mytilus species complex [12,36], which are however limited to small number of markers used to resolve population structures based on allele frequencies.

In this study, we have employed an easy and rapid *de novo* SNP discovery method to develop genome-wide species-specific markers to genotype the *Mytilus* species complex, which also identify hybrids and introgressed individuals in field populations. More accurate characterisation of *Mytilus* specie populations’ structure will aid improved conservation and aquaculture management strategies.

## Materials and methods

### Ethics statement

Animal (mussels) handling and collection was done under Marine Science Scotland (Scottish Government) authority and following both Marine Science Scotland and University of Stirling Ethical recommendations and guidance.

### Sample collection

Adult mussels (at least 40 mm in length) were collected from regions where pure *Mytilus* species were reported to occur, based on historical, genetic analysis or morphological evidence [3,10,11,37,38]. Specimens of *M*. *edulis* were collected from two shoreline locations in southwest Scotland [Loch Ryan (LR) and Rascarrel Bay (RB)] and one shoreline location in east Scotland [Montrose (MON)], the three sites have shellfish farming activities in the vicinities; *M*. *galloprovincialis* were sourced from Slovenia [Bay of Piran (BP)]; and *M*. *trossulus* were acquired from Penn Cove (PC), USA (Table 1). Additional adults *M*. *trossulus* from Bras d’Or Lake (BDL), Canada, and juvenile mussels (approximately 15-months old) from Loch Etive (LET), Scotland were also obtained and used for markers validation purposes (Table 1). Tissue samples (gill/mantle from adults; all body tissues from juveniles) were taken and stored in 99% ethanol at -20°C.

**Table 1:** Details of sampling sites and the number *Mytilus* specimens used for marker discovery and marker validation. * One of the *M*. *trossulus* from Penn Cove was re-assigned as *M*. *edulis* during the marker development/validation stage (see Results). † Additional samples used only for marker validation.

Site location	Site coordinates	Species reported	Me15/16 screening	SNP discovery	KASP validation
LR—Loch Ryan	54°56'06.8"N 5°03'38.7"W	*M*.* edulis*	50	10	50
RB—Rascarrel Bay	54°48'53.1"N 3°51'22.7"W	*M*.* edulis*	50	10	50
BP—Bay of Piran	-	*M*.* galloprovincialis*	50	15	50
PC—Penn Cove	-	*M*.* trossulus*	8	4+1*	8
MON—Montrose	56°42'16.3"N 2°28'13.7"W	*M*.* edulis*	50	-	50
LET—Loch Etive^†^	56°27'05.5"N 5°19'13.3"W	*M*.* trossulus* (juvenal)	20	-	20
BDL—Bras d’Or Lake^†^	45°59'55.4"N 60°43'31.0"W	*M*.* trossulus*	50	-	50

### Me15/16 PCR genotyping

DNA was extracted from tissue and treated with RNase. Each sample was quantified by spectrophotometry (Nanodrop), quality assessed by agarose gel electrophoresis, and stored in 5 mmol/L Tris, pH 8.5. Preliminary PCR were carried out at a single locus with the Me15/16 primer set: Me15: CCAGTATACAAACCTGTGAAGA; Me16: GTTGTCTTAATAGGTTTGTAAGA [29]. Each 6 μL PCR reaction comprised 3 μL 2× MyTaq mix (Bioline); 0.4 μL of 10 μM forward and reverse primer; 0.5 μL template DNA (5–50 ng/μL); and 1.7 μL ultrapure water. PCR conditions were 95°C for 1 min, [95°C for 15 s, 56°C for 15 s, 72°C for 30 s] × 35 cycles. PCR products (1 μL) were run at 60 V for 40 mins on a 2% agarose gel (0.5× TAE, stained with 100 ng/μL EtBr). Using a UV transilluminator, *Mytilus* species and hybrids were identified based on size differentiation of PCR products: 180 bp (*M*. *edulis);* 168 bp (*M*. *trossulus);* 126 bp (*M*. *galloprovincialis)* or a combination in the case of hybrid individuals [29].

### RAD library preparation and sequencing

A total of 40 pure specimens (21 *M*. *edulis*, 15 *M*. *galloprovincialis* and 4 *M*. *trossulus*) were chosen for species reference library construction (S1 Table). The RAD library was prepared as originally described in Baird *et al*. [39] and comprehensively detailed in Etter et al. [40], with minor modifications [41]. Briefly, each sample (0.25 μg DNA) was digested at 37°C for 40 min with the high-fidelity restriction enzyme *Pst*I that recognises the CTGCA|G motif (New England Biolabs; NEB) using 6 U *Pst*I per μg genomic DNA in 1× Reaction Buffer 4 (NEB) at a final concentration of about 1 μg DNA per 50 μL reaction volume. The samples (12 μL final volume) were then heat-inactivated at 65°C for 20 min. Individual specific P1 adapters, each with a unique 5 or 7 bp barcode (S1 Table), were ligated to the *Pst*I digested DNA at 22°C for 15 min by adding 0.6 μL (DNA samples) 100 nmol/L P1 adapter, 0.15 μL 100 mmol/L rATP (Promega), 0.25 μL 10× Reaction Buffer 2 (NEB), 0.125 μL T4 ligase (NEB, 2,000 U/μL) and reaction volumes made up to 15 μL with nuclease-free water for each sample. After heat-inactivation at 65°C for 20 min, the ligation reactions were slowly cooled to room temperature (over 1 h), then combined in appropriate multiplex pools. Shearing (Covaris S2 sonication) and initial size selection (100 to 800 bp) by agarose gel electrophoresis [41] was followed by gel purification, end repair, dA overhang addition, P2 paired-end adapter ligation and library amplification. 120 μL of each amplified library was size-selected (about 250 to 500 bp) by gel electrophoresis. Final libraries were sent to BMR Genomics (Padua, Italy), for quality control and high-throughput sequencing. Libraries were accurately quantified by fluorimetry and calibrated by sequencing on an Illumina MiSeq at the Institute of Aquaculture using 100 base paired-end reads (v3 chemistry). The Libraries were sequenced in four lanes of an Illumina HiSeq 2000, using 100 base paired-end reads (v3 chemistry). Reads were deposited at the European Bioinformatics Institute (EBI) Sequence Read Archive (SRA) study PRJEB7210.

### Genotyping RAD alleles

Reads of low quality (*i*.*e*., with an average quality score less than 20), that lacked the restriction site or had ambiguous barcodes were discarded. Retained reads were sorted into loci and genotypes using Stacks v1.13 [42]. Stacks assigns loci based on nucleotide positions in RAD tags using a likelihood-based algorithm [43] to separate actual SNPs from SNPs likely to have arisen from sequencing error. Using the default parameters for *de novo* assembly pipeline, a minimum stack depth of 5 and a maximum of 2 mismatches were allowed per locus in an individual, with no more than 1 mismatch between alleles. Informative RAD markers were kept only when presenting a maximum of three SNPs and one to three alleles present in all three species and at least 50% of the samples. Diagnostic species-specific markers were Informative RAD markers with a fixed allele within species but presenting different allele between at least two of the three species.

### Phylogenetic analysis

Sequencing data from filtered Informative RAD markers was combined into a single alignment of alleles (composite genotype) for a total of 40 individuals used in RAD library construction. Phylogenetic trees were constructed with RAxML (Randomised Axelerated Maximum Likelihood), using the RAxML v8.0.0 [44]. Maximum-likelihood phylogenetic trees were inferred using the GTR+CAT nucleotide substitution model [45] and bootstrap support values estimated from 10,000 replicate searches of randomly generated trees.

### Data analysis

Data analysis was carried out using R v3.3.2 [46] and an associated R/*adegenet* package v1.4–1 [47] for Principal Component Analysis (PCA) and Discriminant Analysis of Principal Components (DAPC). PCA creates simplified models of the total variation within the dataset and DAPC identifies clusters of genetically related individuals [48].

### SNP-assay design

Each tested locus comprised two alleles that were identifiable by the presence of a SNP. One allele was diagnostic for a single species, while the other allele was shared by the remaining two species. For primer design to be feasible, the SNP of interest at a given locus needed to be at least 20 bp from the end of a given sequence. This allowed enough sequence for compatible primers to be designed. SNP assays were designed and manufactured for use with KASP genotyping technology by LGC Genomics Ltd. (S2 Table). Each sample was genotyped in 5 μL reactions each containing approximately 40 ng template DNA. Optimisation assay conditions were 94°C for 15 min; [94°C for 20 s, 61–55°C for 120 s (0.6°C drop per cycle)] × 10; and [94°C for 20 s, 55°C for 120 s] × 40. Each 5 μL reaction comprised 2.5 μL 2× KASP Master Mix; 0.07 μL KASP Assay Mix; 0.4 μL template DNA (minimum concentration of 5 ng/μL); plus 2.1 μL ultrapure water. An addition of 0.25 100% DMSO was added for markers G4, T4 and T5. Individual genotype assignment was performed through reading the fluorescence emission of the FAM and HEX fluorophores for each sample, in comparison to no-template control reactions, using a Techne Quantica Real Time PCR Thermal Cycler and Quansoft endpoint genotyping software (Bibby Scientific).

### Population structure

Structure v2.3 [49] was used to identify distinct genetic populations from multilocus (SNP) data, assigning individuals to populations, and identifying admixed individuals. The “Admixture Model” was used assuming that each genotyped individual could have mixed ancestry, inheriting some fraction of its genome from ancestors in a different population. This would be an assumption made for reference populations of pure species, while the other could have migrants that have interbred with native individuals.

## Results

### Me15/16 pre-evaluation

Putative pure *Mytilus* species individuals collected in locations where only pure species were previously reported [*M*. *edulis* (Loch Ryan and Rascarrel Bay); *M*. *galloprovincialis* (Bay of Piran); and *M*. *trossulus* (Penn Cove)] were screened using the Me15/16 locus (Table 2 and S3 Table) to confirm their genotype. A total of 40 individuals, genotyped as pure (homozygous) with Me15/16 were chosen for RAD library construction: these included of 21 *M*. *edulis* (10 each from Loch Ryan and Rascarrel Bay and a single individual from Penn Cove); 15 *M*. *galloprovincialis*; and four *M*. *trossulus* from Penn Cove. *M*. *trossulus* produced a small sample size because of the very limited DNA material available for this species.

**Table 2:** Summary results the preliminary Me15/16 genotyping. Genotypes are as follows: *M*. *edulis* [Me]; *M*. *galloprovincialis* [Mg]; *M*. *trossulus* [Mt]. Reported numbers are number of individual presenting a given genotype. Hybrid are shown as composites. Site names are abbreviated as detailed in Table 1.

Site	Me	Mg	Mt	Me/Mg	Me/Mt	Mg/Mt
LR	49	0	0	1	0	0
RB	50	0	0	0	0	0
MON	48	0	0	2	0	0
BP	0	50	0	0	0	0
PC	1	0	7	0	0	0
BDL	0	0	40	0	10	0
LET	0	0	20	0	0	0

### RAD library sequencing

High throughput sequencing of these 40 individuals produced 574,728,488 raw reads in total (four HiSeq lanes and one MiSeq lane). MiSeq technology was used to adjust the libraries. After the removal of low-quality and incomplete reads, 71.9% of the total raw reads were retained (413,377,018 reads). As only *M*. *galloprovincialis* has a published draft genome (NCBI assembly GCA_001676915.1) of over 1 million contigs and that only 35% of the reads from *M*. *galloprovincialis* samples were aligned to it, a *de novo* approached was used to assemble the RAD tags. A total of 3,253,798 RAD tags were detected (S1 Table).

### Sequence analysis

The number of RAD tag detected per individual was relatively consistent, ranging from 59,000–313,000 RAD tag (Table 1). There were two exceptions among *M*. *edulis* individuals with significantly lower number of tags obtained (RB_01 and PC_01, which had 18,220 and 5,459 RAD tags respectively) which was most likely caused by low quality DNA resulting in effecting the library preparation efficiency. Between 14% and 15% of the RAD tags were polymorphic. To identify robust genetic markers and to minimise the proportion of erroneous data, all informative markers were filtered to show only those with one to three alleles and a maximum of three SNPs, and which were detected in all three species and at least 50% of the samples. A total of 14,212 SNPs spread across 6,220 informative RAD markers were identified (some loci had more than one SNP), and used in subsequent analyses. A reduced set of markers, 378 SNPs spread across 365 diagnostics species-specific markers, were filtered out as the informative RAD markers when exhibiting fixed allele within species, but presenting different allele between at least two of the three species (S4 Table).

### Phylogenetic reconstruction

**Fig 1:**
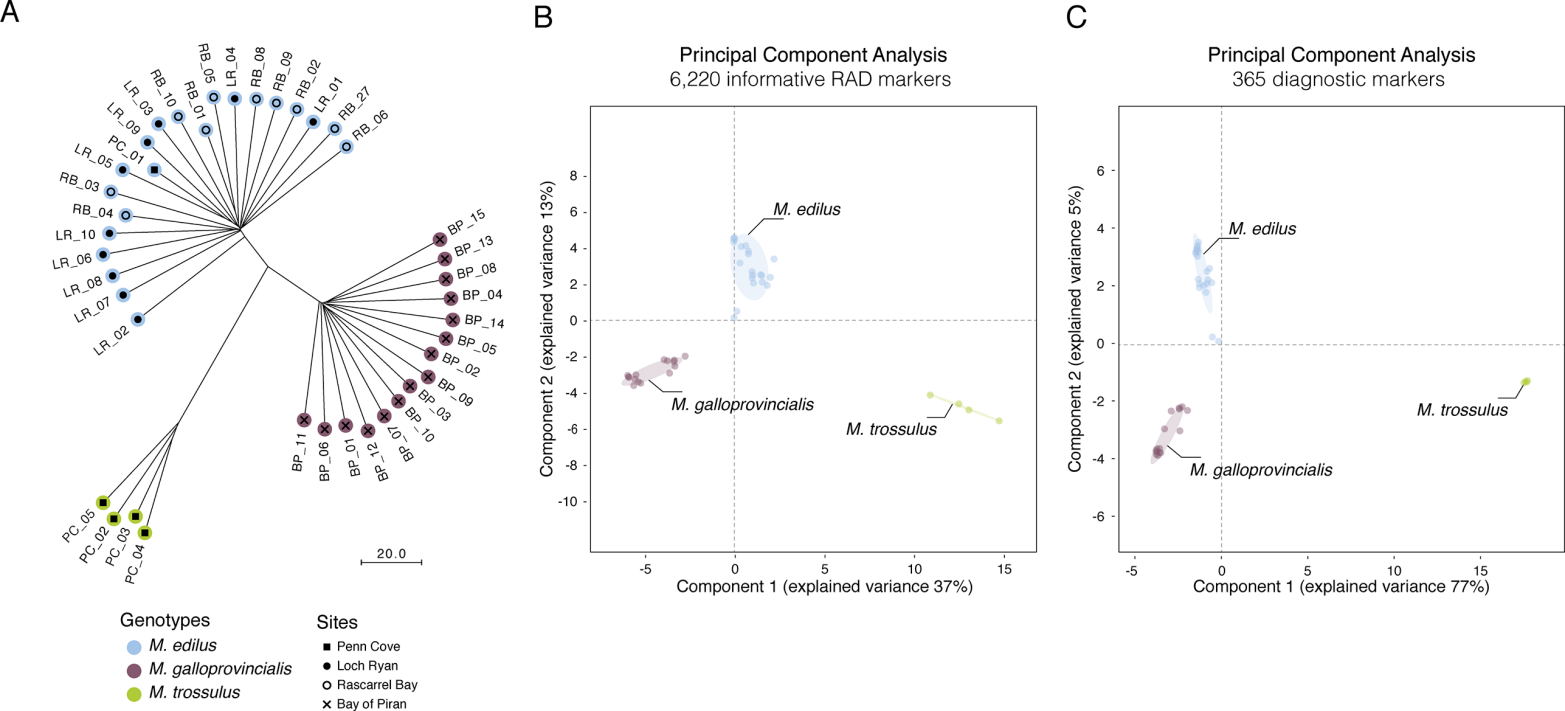
Capture the discriminant ability of RAD markers. (A) Phylogenetic reconstruction based on the SNP of the 6,220 informative RAD markers (RAxML). Genotype were established using preliminary Me15/16 PCR assay. The scale shows the number of nucleotide substitutions per site (B) Principal Component Analysis of the 6,220 informative RAD markers. (C) Principal Component Analysis of the 365 diagnostic markers. RB_01 and PC_01 while grouping with *M*. *edulis* exhibit lower polymorphism due to the highest number of missing data compared to all other samples.

The phylogenetic tree constructed from the composite genotypes of 6,220 informative RAD markers shared alleles (14,212 SNPs) showed three distinct clusters, accurately delineating the three species (3 sites for *M*. *edulis*, one site for *M*. *galloprovincialis* and *M*. *trossulus*) that were used for library construction (Fig 1A). *M*. *edulis* and *M*. *galloprovincialis* were the closest (a genetic distance of 33 nucleotide substitutions between the base *M*. *edulis* and *M*. *galloprovincialis* branches) and *M*. *trossulus* was more distant (a genetic distance of 79 nucleotide substitutions). One *M*. *edulis* from Penn Cove was grouped with the *M*. *edulis* from Loch Ryan and Rascarrel Bay, confirming its identity as *M*. *edulis* as suggested by the Me15/16 genotyping.

### Marker selection

In order to better capture the *Mytilus* species complex structure the ability of each marker to discriminates each “pure” species, a principal component analysis (PCA) was conducted from 6,220 informative RAD markers using *R/adegene*t (Fig 1B). Three distinct clusters were separated using the first two components (88.7% of cumulative variance) despite the small number of samples examined.

A second PCA was applied on the reduced set of 365 diagnostics species-specific markers, to ensure that they kept their discrimination power (Fig 1C). Subsequently, the Discriminant Analysis of Principal Components (DAPC) sorted species diagnostic loci by their “loading values”, values based on the population coverage at each locus [47]. Loci with the highest presence had the highest loading values and, thus, were assumed to be less likely to be false positive, improving their reliability as potential diagnostic markers. Loci with the highest “loading values” were preferred; as long as they matched the other selection criteria for KASP assay development.

### SNP assay optimisation

All SNP assays were designed for use with KASP genotyping technology by LGC Genomics Ltd. Assay optimisation was carried out with the 40 samples used in RAD library construction. A total of 12 SNP assays, three assays per *Mytilus* species, were successfully optimised (S2 Table): E1, E2 and E3 (*M*. *edulis*); G1, G2, G3 and G4 (*M*. *galloprovincialis*); and T1, T2, T3, T4 and T5 (*M*. *trossulus*). SNP genotyping results (KASP and RAD) were obtainable at all 12 loci for each of the 40 samples (S5 Table) revealing identical results regardless of the genotying technique.

### SNP assay validation

**Fig 2:**
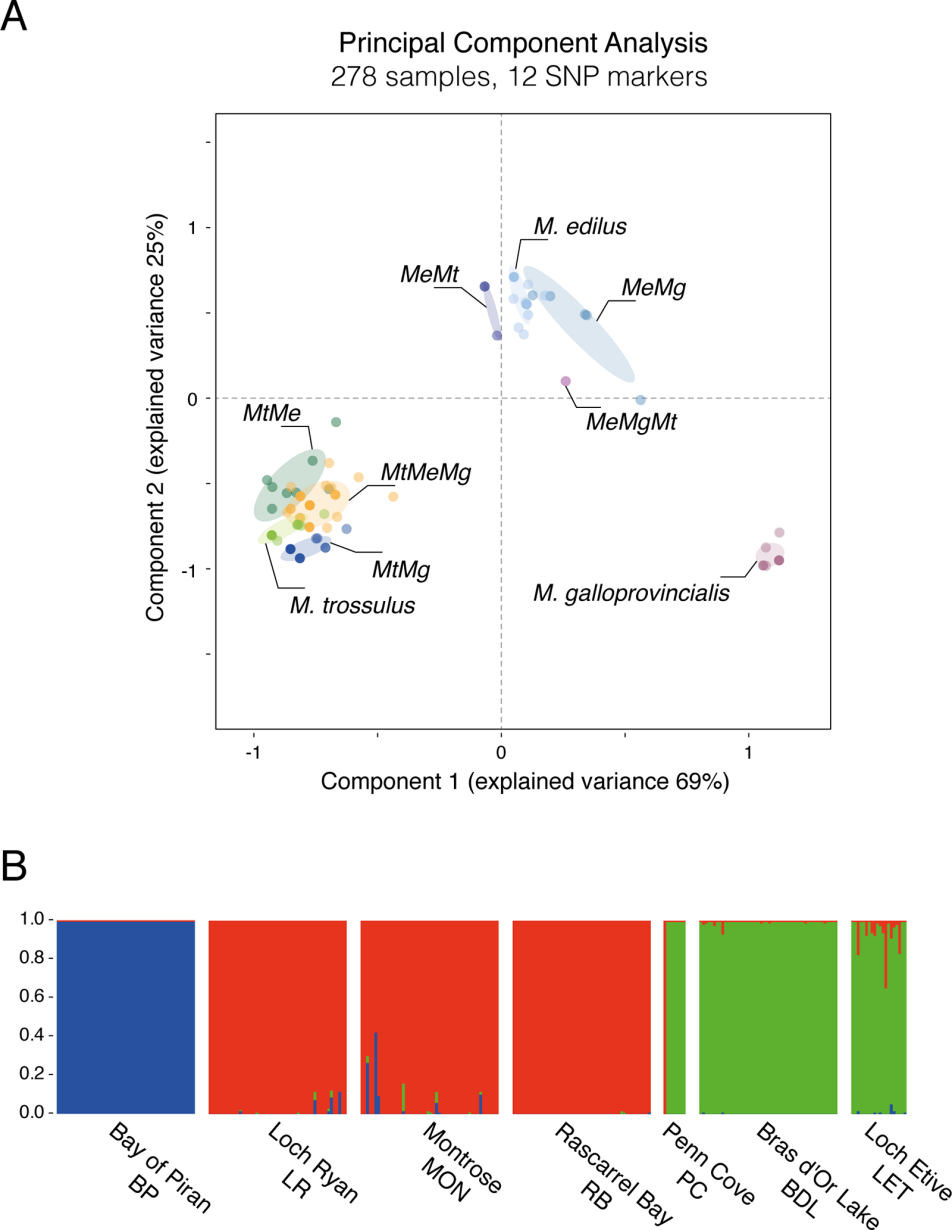
Multilocus genotyping across 12 SNP makers. (A) Principal Component Analysis of the 12 diagnostic markers across the 278 samples. The Genotype classes are clustered and annotated on the figure. (B) Structure plots constructed using the Admixture Ancestry Model with independent allele frequencies per population (K = 3, burnin = 10,000, reps = 100,000), showing the genetic composition of reference and validation population.

238 samples, including 150 samples from 3 additional populations, were genotyped with the 12 SNP assays (Table 3 and S6 Table). Additional populations were sourced in order to validate the markers with individuals from different origins, thereby reducing the risk of developing markers that would be population- or location-specific rather than species-specific. Where all diagnostic alleles were attributed to only one species, individuals were identified as “pure” species (*M*. *edulis* [Me], *M*. *galloprovincialis* [Mg] or *M*. *trossulus* [Mt]). Individuals heterozygote at all diagnostic loci for two species would be identified as F1 hybrids; however, none were found in any of the population sampled. All other individuals were identified as introgressed individuals. Principal Component Analysis (PCA) discriminated the three “pure” species while the introgressed individuals were intermediates with respect to their genetic background (Fig 2A). Based on their position, the dominating background and one or more other species influencing the genetic make-up can be estimated.

**Table 3:** Summary results the 12 SNP assay. Genotypes are as follows: *M*. *edulis* [Me]; *M*. *galloprovincialis* [Mg]; *M*. *trossulus* [Mt]; Hybrids are shown as composites. Site names are abbreviated as detailed in Table 1.

Site	Me	Mg	Mt	MeMg	MeMt	MtMg	MeMgMt
LR	48	0	0	2	0	0	0
RB	49	0	0	0	1	0	0
MON	44	0	0	3	2	0	1
BP	0	50	0	0	0	0	0
PC	1	0	7	0	0	0	0
BDL	0	0	12	0	5	10	23
LET	0	0	5	0	6	4	5

Structure modelling showed three distinct clusters of genotypes; this model best corresponded to three distinct genotypes (*M*. *edulis*, *M*. *galloprovincialis* and *M*. *trossulus*) in the populations studied (Fig 2B). The four populations used for RADseq were suitable pure populations. These models suggest that, despite the introgression observable with the SNP genotyping, these populations are mostly pure with some mixing and thus were suitable for diagnostic marker design.

**Fig 3:**
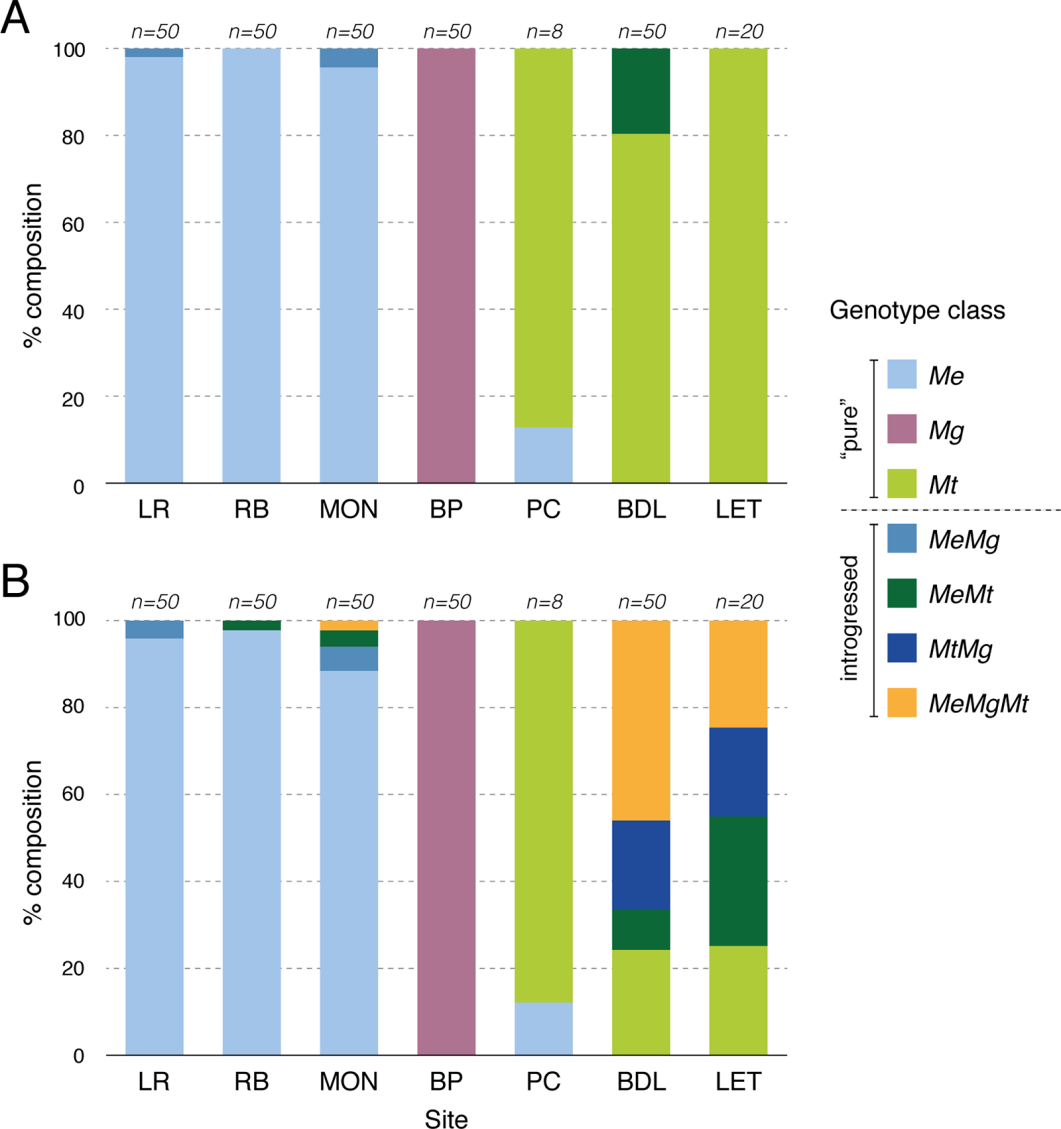
Genotype classes identified in seven populations. (A) Single locus Me15/16 (details in Table 2). (B) Multilocus genotyping across 12 SNP markers. No F1 hybrid was detected (details in Table 3).

Bay of Piran (100%; 50/50), Penn Cove (100%; 8/8), Rascarrel Bay (98%; 49/50), and Loch Ryan (96%; 48/50) had the highest proportions of pure individuals, followed by Montrose (88%; 44/50). Bras d’Or Lake (24%; 12/50) and Loch Etive (25%; 5/20) showed a high proportion of introgressed individuals; Both location have been reported as having hybrids *M*. *edulis* × *M*. *trossulus* [10,37]. Loch Etive population was expected to have 100% *M*. *trossulus* individuals according to the Me15/16 locus genotyping (Fig 3A); however, the SNP analysis revealed that only 25% were “pure” *M*. *trossulus*, and the other 75% of the individuals were hybrids with various degrees of introgression with *M*. *edulis* and/or *M*. *galloprovincialis* (Fig 3B).

## Discussion

Historically, there has been much debate on the taxonomic status of species belonging to the *Mytilus* species complex (*M*. *edulis*, *M*. *galloprovincialis* and *M*. *trossulus*), and whether they are discrete species. Species diagnostic marker development could only take place if three discrete species were present; thus, assessing the phylogenetic relationships of the populations used for diagnostic marker development was crucial for this research. Indeed, Fig 1 shows a clear separation of individuals based on genotype and not population, multiple sites were used for *M*. *edulis*. As confirmed by the KASP assays on the same dataset (Fig 2), all diagnostic markers appeared fixed in the putative “pure” populations, despite the limitation caused by the small number of *M*. *trossulus* specimens (only 4 samples); However, both phylogenetic tree and PCA clearly distinct three groups, consistent with the three species. Furthermore, the identification of 6,220 informative RAD markers and the development of 12 diagnostics markers (chosen out of the 365-possible identified) is a unique opportunity to enhance understanding of the genotype for the application of basic physiological studies and to understand the biological differences between *M*. *trossulus*, *M*. *edulis* and *M*. *galloprovincialis*. The informative RAD markers (polymorphic marker present in at least 50% of the samples) and diagnostic markers (Informative RAD markers with fixed allele within species but presenting different allele between at least two of the three species) identified in this study can be used for many purposed: bio-diversity evaluation, phylogenetic studies, species and population identification. This enhanced understanding of the genetic structure within and between populations, also provides opportunities to better clarify the relationship between genotype and phenotype, particularly in sympatric populations.

Within the aquaculture context, the availability of this new suit of diagnostic markers can allow the sourcing of seeds of known genotypes and the selection of seeds of desirable genotypes for broodstock for ongoing efforts into the hatchery production of seeds. This will significantly contribute to the future eradication of potentially economically damaging species. Improved stock management and potential selective breeding will result in superior resilience and increased productivity of the mussel’s aquaculture industry. Indeed, with the application of multilocus genotyping on Scottish mussels, we have identified introgressed genotypes that were hitherto unrecognisable by using single locus (Me15/16) genotyping. By doing so we improved our understanding of the genetic diversity within and between populations currently present in farmed and wild populations along the Scottish coast. This quick and relatively in-expensive methodology can be extended to any species complex where the phenotype does not provide conclusive evidence for species assignment and where the genetic structure is unknown or poorly established.

## Supporting information

S1 Table Sample and RAD barcodes. Details each sample used: sample ID, library number, Me15/16 Genotype, RAD barcode (P1 adapter), RAD barcode (P1 adapter), number of raw reads (paired-ended) and number of RAD-tags.
(CSV)

S2 Table KASP assay primer sequences. List of the allele-specific primers and common primer designed for the allele-specific PCR genotyping assay.
(CSV)

S3 Table Details of the Me15/16 genotyping results. Genotypes results for Me15/16 preliminary assay for all 278 samples, summarised Table 2.
(CSV)

S4 Table Details of the 378 selected SNP markers. N means no SNP reported for the species. To be selected a SNP needs to be present in at least 50% of all samples and to be reported in a species, the SNP needs to be in at least 50% of the samples of this species (see Materials and Methods).
(CSV)

S5 Table Details of the RAD and KASP assay results. Genotypes of the 12 assays for 40 development individuals.
(CSV)

S6 Table Details of the KASP genotyping results. Genotypes results for the 12 KASP assays for all 278 samples.
(CSV)

## Abbreviations

SNP: Single-Nucleotide Polymorphism
RAD: Restriction site Associated DNA
PCA: principal component analysis
KASP: Kompetitive Allele Specific PCR
